# Molecular Mechanism of the Effect of Huanglian Jiedu Decoction on Type 2 Diabetes Mellitus Based on Network Pharmacology and Molecular Docking

**DOI:** 10.1155/2020/5273914

**Published:** 2020-10-19

**Authors:** Bei Yin, Yi-Ming Bi, Guan-Jie Fan, Ya-Qing Xia

**Affiliations:** ^1^School of Second Clinical Medicine, Guangzhou University of Chinese Medicine, Guangzhou, China; ^2^Department of Endocrinology, Guangdong Provincial Hospital of Chinese Medicine, The Second Affiliated Hospital of Guangzhou University of Chinese Medicine, Guangzhou, China

## Abstract

**Background:**

Huanglian Jiedu Decoction (HLJDD) is a Traditional Chinese Medicine (TCM) formula comprising four herbal medicines. This decoction has long been used in China for clinically treating T2DM. However, the molecular mechanism of HLJDD treat for T2DM is still not fully known. Hence, this study was designed to reveal the synergistic mechanism of HLJDD formula in the treatment of T2DM by using network pharmacology method and molecular docking.

**Methods:**

Retrieving and screening of active components of different herbs in HLJDD and corresponding T2DM-related target genes across multiple databases. Subsequently, STRING and Cytoscape were applied to analysis and construct PPI network. In addition, cluster and topological analysis were employed for the analysis of PPI networks. Then, the GO and KEGG enrichment analysis were performed by using ClueGO tool. Finally, the differentially expressed analysis was used to verify whether the expression of key target genes in T2DM and non-T2DM samples was statistically significant, and the binding capacity between active components and key targets was validated by molecular docking using AutoDock.

**Results:**

There are 65 active components involved in 197 T2DM-related targets that are identified in HLJDD formula. What is more, 39 key targets (AKT1, IL-6, FOS, VEGFA, CASP3, etc.) and 3 clusters were obtained after topological and cluster analysis. Further, GO and KEGG analysis showed that HLJDD may play an important role in treating T2DM and its complications by synergistically regulating many biological processes and pathways which participated in signaling transduction, inflammatory response, apoptotic process, and vascular processes. Differentially expressed analysis showed that AKT1, IL-6, and FOS were upregulated in T2DM samples and a significant between sample differential expression. These results were validated by molecular docking, which identified 5 high-affinity active components in HLJDD, including quercetin, wogonin, baicalein, kaempferol, and oroxylin A.

**Conclusion:**

Our research firstly revealed the basic pharmacological effects and relevant mechanisms of the HLJDD in the treatment of T2DM and its complications. The prediction results might facilitate the development of HLJDD or its active compounds as alternative therapy for T2DM. However, more pharmacological experiments should be performed for verification.

## 1. Introduction

Type 2 diabetes mellitus (T2DM) is a metabolic disease caused by impaired insulin secretion or insulin resistance, which induces glucose, fat, and protein metabolism disorders in the body. T2DM is often associated with a high risk of microvascular complications (including retinopathy, nephropathy, and neuropathy) and macrovascular complications (such as cardiovascular complications) [1]. The prevalence of T2DM is ever-increasing with years [2]. According to statistics, in 2015, 415 million adults aged 20-79 years around the world had diabetes (more than 90% are T2DM), and China ranked first (109.6 million) [3]. The IDF report estimates that the number of deaths due to diabetes and its complications increased to 5 million during 2015, which is equivalent to one death every six seconds [4]. The epidemic of diabetes and its complications pose a major threat to global health, greatly increasing the burden of death and disability. However, the current mainstream treatments for T2DM mainly include diet control, exercise, and oral administration glycemic drugs, and subcutaneous injection of insulin shows limited efficacy. The therapeutic mechanism is focused on a single component, which is effectively control the blood glucose level of patients, but it has no preventive effect on complications. Therefore, based on the advantages of multicomponent and multitarget effects of Traditional Chinese Medicine (TCM), finding a multitarget hypoglycemic drug from TCM resources has gradually become a new research hotspot.

Huanglian Jiedu Decoction (HLJDD) comes from the classic book “The Handbook of Prescriptions for Emergencies” of TCM; it is the first clinical emergency manual in China, which consists of four herbs, namely, Coptidis rhizome, Scutellariae radix, Phellodendri chinensis cortex, and Gardeniae fructus, known as Huanglian (HL), Huangqin (HQ), Huangbo (HB), and Zhizi (ZZ) in Chinese, in the ratio of 3 : 2 :  2 : 3, respectively. It is a heat-clearing agent with the effect of clearing heat and detoxifying. Many scholars have conducted research on the efficacy of HLJDD in the treatment of T2DM. Two meta-analytical papers in China have shown that HLJDD alone is equivalent to metformin in treating T2DM; adding HLJDD to basic hypoglycemic treatment can effectively reduce the levels of Hb1Ac and 2hPG in T2DM patients and can be used to treat early HbAlc < 7.3% T2DM patients [5, 6]. Zhang et al. found that HLJDD had a significant effect on controlling blood glucose levels and weight and also have a lipid-regulating effect in T2DM rats [7]. However, due to the complex composition of HLJDD, the relevant mechanism for treating T2DM has not been fully elucidated.

**Figure 1:**
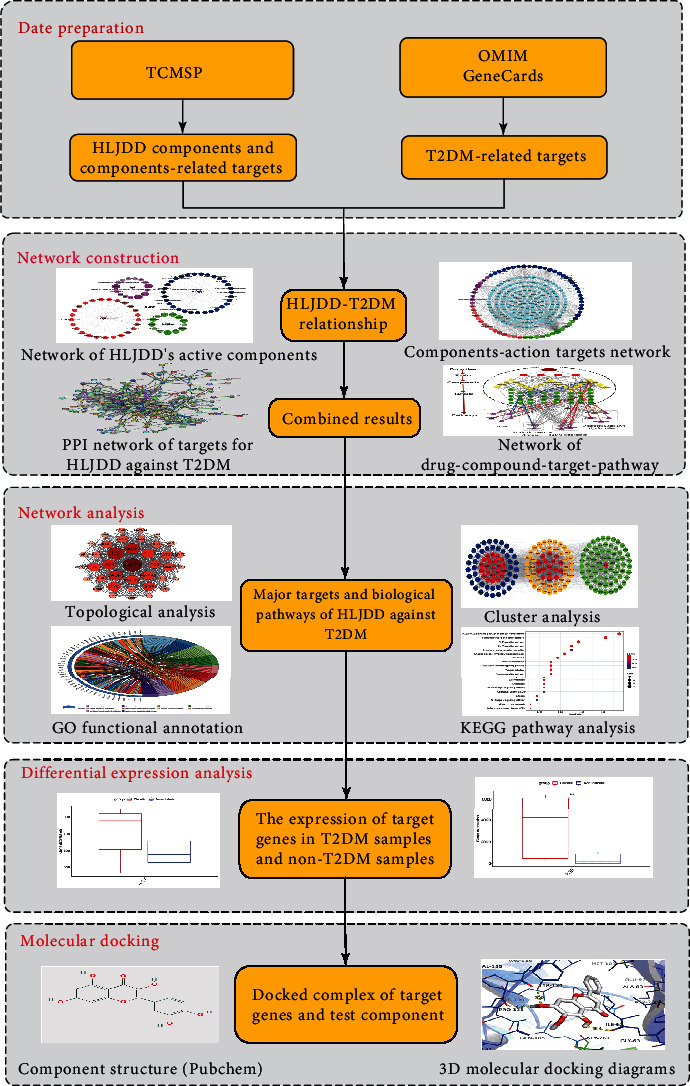
Workflow for HLJDD in the treatment of T2DM.

Network pharmacology uses computers, high-throughput omics data analysis, and other technologies to deeply study the pharmacological mechanism of the role of TCM; its research strategy is holistic and systematic, which is consistent with the overall concept of TCM and the principles of dialectical treatment [8]. Our study is the first to use the network pharmacology method to screen the effective bioactive ingredients and targets of HLJDD, analyze its key targets, and signal pathways for the treatment of T2DM, in order to provide a guideline in the further investigation of this formula. The detailed workflow of this study was showed in Figure 1.

## 2. Materials and Methods

### 2.1. Materials

See (Table 1).

### 2.2. Methods

#### 2.2.1. Collection and Screening of Bioactive Compounds in HLJDD

All candidate herbal compounds of HLJDD were harvested by use of Traditional Chinese Medicine Systems Pharmacology Database and Analysis Platform (TCMSP). Based on literature reports and pharmacokinetic parameters, the pharmacokinetic properties including absorption, distribution, metabolism, and excretion (ADME) are important contributors for bioactivities of drug. As the TCMSP suggested, the compounds with oral bioavailability (OB) ≥ 30% have good absorption and slow metabolism after oral administration. The compounds with drug − likeness (DL) ≥ 0.18 were chemically suitable for drug development. Hence, two ADME-related parameters including OB ≥ 30% and DL ≥ 0.18 were employed to identify the potential active compounds in HLJDD [8].

#### 2.2.2. Prediction of Compounds-Related Targets

Thereafter, we undertook a search for targets that corresponding to the active components of HLJDD obtained from the TCMSP. Use UniProt database to convert all target proteins into corresponding gene symbols with “Homo sapiens” species to standardize gene names and organisms, which prevents overannotation of similar proteins [9]. Finally, the information of compound targets was obtained.

#### 2.2.3. Identification of T2DM-Related Targets and Predicting Therapeutic Targets of T2DM

Details on the human genes associated with T2DM were screened from the GeneCards and OMIM database. Using “T2DM” and “type 2 diabetes mellitus” as keywords, and only “Homo sapiens” genes linked to T2DM were acquired. All the target genes obtained above two databases were summarized. Subsequently, active compound targets were mapped to T2DM-related targets, and then, therapeutic targets of HLJDD against T2DM were obtained by using a Venny2.1 online tool.

#### 2.2.4. Protein–Protein Interaction (PPI) Data

The Search Tool for the Retrieval of Interacting Genes (STRING) database provides information on predicted and experimental protein interactions. Enter the therapeutic targets into the String database as a gene symbol. Furthermore, the condition was limited to “Homo sapiens,” and the free point was hidden. In the present study, a high confidence score with higher than 0.9 was selected to obtain a PPI data and then save the tsv file for further research.

#### 2.2.5. Network Construction and Analysis

Network construction was performed as follows: (1) the HLJD decoction-active compound network was established by connecting the herbs of HLJDD and their corresponding active compounds; (2) the active compounds-targets network of HLJDD was established by connecting the active compounds and their related targets; (3) a PPI network of therapeutic targets for HLJDD against T2DM was constructed; (4) network of key targets for HLJDD against T2DM by topological analysis was built; (5) clustering modules in the PPI network was built; and (6) decoction–compound–target–pathway network was constructed. All the networks were visualized utilizing by the Cytoscape v3.7.2 software, which is a useful tool for analysis and visualization of molecular interaction networks.

In order to pick out key target genes with high degree of connectivity for HLJDD against T2DM, we use a CytoNCA tool (a plug-in of Cytoscape) to analyze the topological properties of targets. There are four parameters including degree centrality (DC), betweenness centrality (BC), closeness centrality (CC), and eigenvector centrality (EC) were used to estimate the central properties of the nodes in the network. The higher the four quantitative values of a node are, the greater the importance of the node in the network. In the PPI network, DC greater than 2 times the median, BC, CC, and EC are greater than 1 time the median were employed to screen the key targets of HLJDD acting on T2DM.

Targets in different clusters may play similar physiological roles, thus affecting human physiological and pathological processes. To obtain the links between network clusters, a Cluster Marker tool (a plug-in of Cytoscape) was adopted to investigate node composition in the PPI network.

#### 2.2.6. Gene Ontology (GO) Functional Annotation and Kyoto Encyclopedia of Genes and Genomes (KEGG) Pathway Analysis

To elucidate the biological processes of the target proteins in different clusters and their role in signaling transduction, the ClueGO tool (a plug-in of Cytoscape) is used to perform GO functional annotation and KEGG pathway analysis. By entering a list of target gene names and limiting the species to be human, all target gene names are corrected to their official gene symbol. GO terms with *p* value < 0.01 and KEGG pathways with *p* value < 0.01 corrected by Bonferroni method were considered to have significance. Finally, choosing the first 20 entries and draw them into a bubble chart for visualization by using the online Omishare website.

#### 2.2.7. Differentially Expressed Analysis

The gene expression profiles associated with T2DM were obtained from the Gene Expression Omnibus (GEO) database. The following search terms were used: “Type 2 diabetic or T2DM or Type 2 Diabetes Mellitus,” “Homo sapiens,” and “Expression profiling by array.” After careful screening, the microarray data (GSE29231) submitted by Jain et al. were selected for subsequent analysis [10]. The experimental platform GPL6947 used in the above study was the Illumina HumanHT-12 v3 Expression BeadChip arrays. The dataset included a total of 24 samples: 12 T2DM visceral adipose female samples and 12 non-T2DM visceral adipose female samples. Then, verify whether the targets with highest DC in different clusters are differentially expressed between T2DM and non-T2DM samples using differentially expressed analysis, *P* < 0.05 was considered statistically significant. Finally, use the box plots to show the specific expression of the target genes. Since gene expression profiles are downloaded from GEO (a public database), we do not need to ethically approve, and we do not conduct new experiments on patients or animals.

#### 2.2.8. Component-Target Molecular Docking

AutoDock 4.2.6 is used as molecular docking software for semiflexible docking [11]. The specific operations are as follows: (1) ligand molecule preparation. Determine the compound name, molecular weight, and 2D structure of the compound from the PubChem database. The 3D structure of the compound constructed with the ChemOffice software is saved in ∗ mol2 format. (2) Receptor molecule preparation, select the targets with highest DC in different clusters as the target protein and obtain the 3D structure of the protein receptor from the RCSB PDB database in ∗ PDB format. Use the Pymol 2.3 software to extract the original ligand conformation of the target protein and save it in PDB format. (3) Molecular docking, add polar hydrogen and Gasteiger charge to the processed receptors and ligands with AutoDock Tools (ADT) and save the processed receptor and ligand files in PDBQT format. Next, use AutoGrid tool to set the parameters of the docking box (adjust the X-Y-Z coordinates and grid size), apply the Lamarckian genetic algorithm (LGA) to find the best docking conditions for flexible docking, and record the docking position of the receptor and ligand. The analysis of molecular docking results refers to the binding energy (*Δ*Gbind). If the binding energy is less than -5 kJ∙mol^−1^, it indicated that the target has certain binding activity with the compound [12]. The lower the binding energy, the better the docking effect. Finally, use the Pymol software to analyze and observe the docking results of compounds and proteins.

## 3. Results

### 3.1. HLJD Decoction-Active Compounds Network Analysis

**Figure 2:**
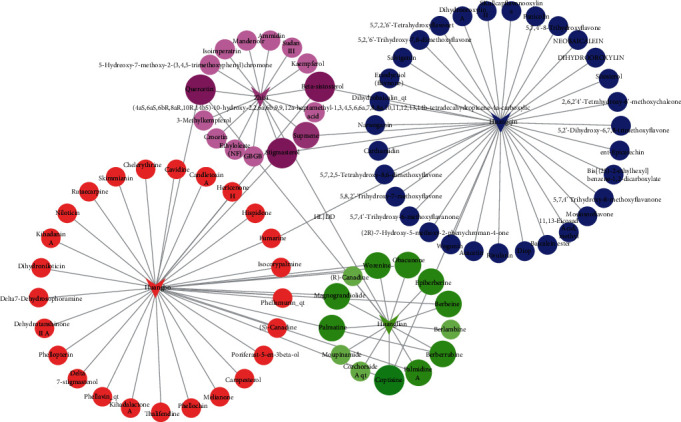
Network of HLJDD's active components. HLJDD formula is consists of HL, HQ, HB, and ZZ. V corresponds to 4 herb nodes. Green, dark blue, red, and purple circles employed to stand for compounds of HL, HQ, HB, and ZZ, respectively. Node size of the compound is proportional to the number of Chinese medicines connected, indicating that different Chinese medicines contain the same chemical components.

By searching the TCMSP databases, the compounds contained in HL, HQ, HB, and ZZ are 48, 143, 140, and 98, respectively. According to the characteristics of OB and DL of the compounds, 14, 36, 37, and 15 active ingredients were screened out, respectively. There are 89 nodes (84 compound nodes, 4 herb nodes, and 1 formula nodes) and 105 edges composed HLJD decoction-active compounds network (Figure 2). In this network, coptisine and berberine exists in HL, HQ, and HB. Quercetin exists in HL, HB, and ZZ. Stigmasterol and beta-sitosterol exist in HQ, HB, and ZZ. Supraene exists in HQ and ZZ. Berberrubine, palmidin A, magnograndiolide, worenine, and obacunone exist in HL and HB. The results indicate that the same components may exist in multiple Chinese medicines, and different medicines contain multiple effective components which is an important material basis for the multiple target effects of TCM.

### 3.2. Active Compounds-Targets Analysis

**Figure 3:**
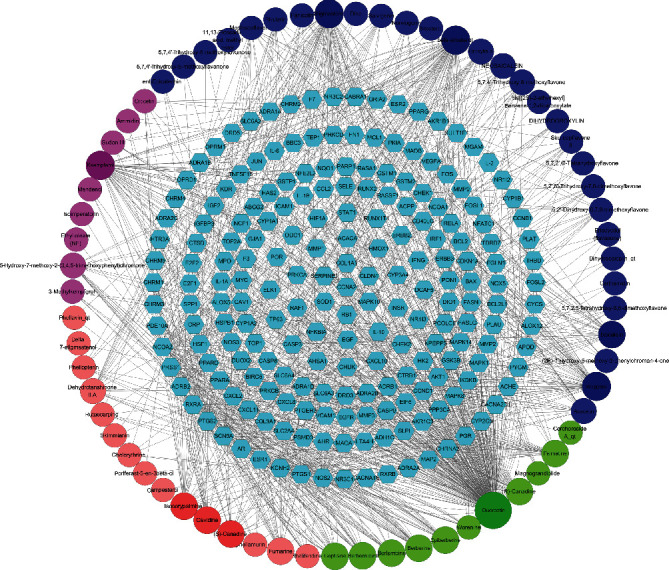
Network of active components-targets. 203 human-derived target proteins target nodes represented by light blue hexagon. Green, dark blue, red, and purple circles employed to stand for compounds of HL, HQ, HB, and ZZ, respectively.

The corresponding targets of HL, HQ, HB, and ZZ were 133, 88, 55, and 170, respectively. After removing duplicate targets, a total of 203 human-derived target proteins were obtained. The active compounds- targets network of HLJDD is shown in Figure 3. The network consists of 268 nodes (203 target nodes and 66 compound nodes) and 1074 edges, where the nodes represent the active compounds of herbs and compound-related targets, and the edges represent the interaction between the active compounds and the target protein. Among these targets, a total of 135 targets involved in quercetin, 51 targets related to wogonin and kaempferol, and 29 targets involved in baicalein. Other active compounds also have more corresponding targets, such as isocorypalmine, beta-sitosterol, and stigmasterol, all correspond to 26 targets. This suggests that compounds in the HLJDD formula may work together on these targets, exerting pharmacological effects in T2DM and other diseases.

### 3.3. PPI Network of Therapeutic Targets for HLJDD against T2DM

**Figure 4:**
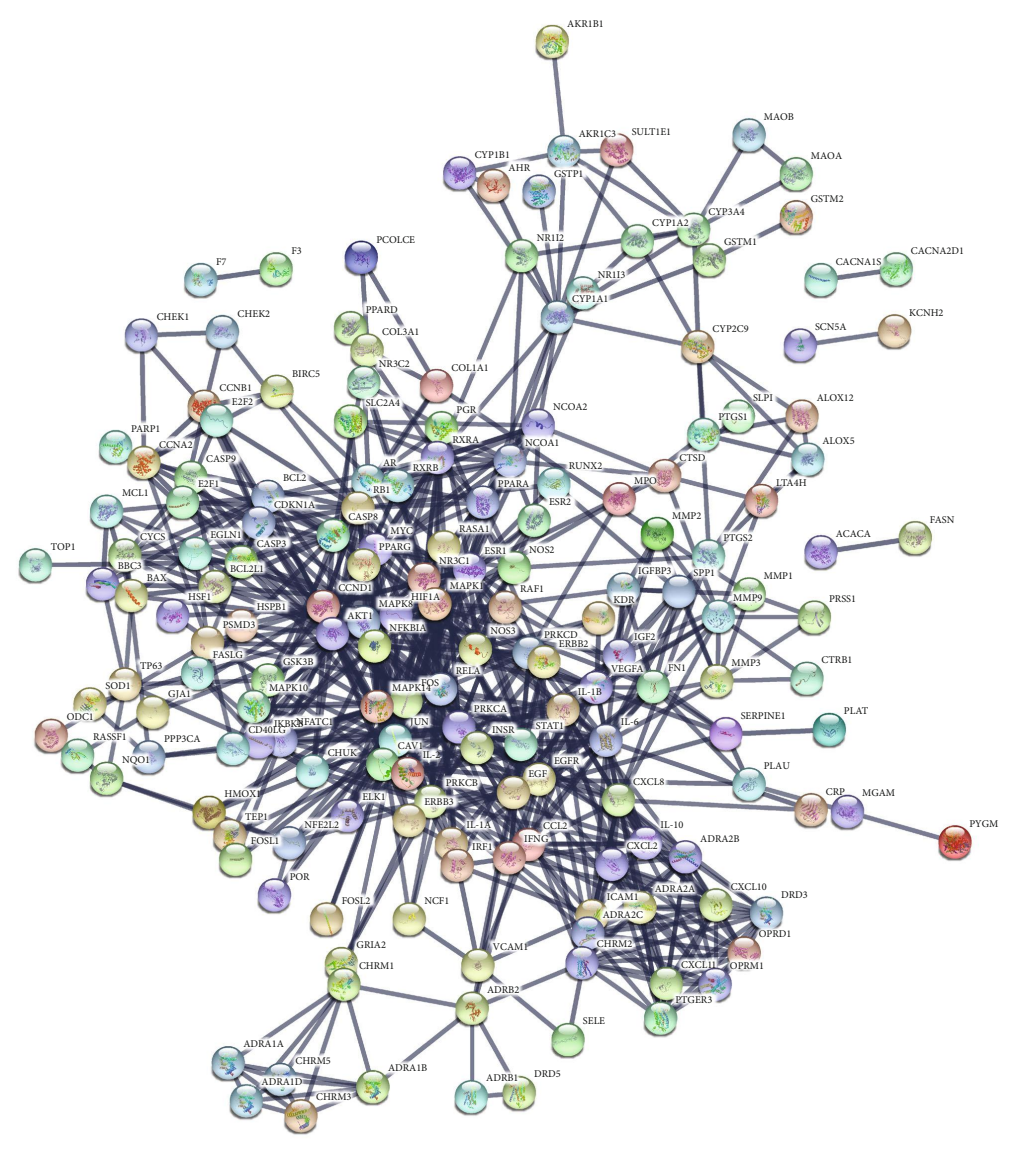
PPI network of targets for HLJDD against T2DM.

From the GeneCards and OMIM database, 11,230 T2DM-related targets were acquired. A total of 65 active components (Table 2) and 197 overlapping genes were obtained by looking for the intersection of the 203 compound-related targets and the 11,230 T2DM-related targets by using Venny2.1 online tool. The 197 overlapping genes were submitted to the STRING database. At the same time, hide the free point (DCAF5 and DIO1). Finally, 195 target genes with higher degree (degree ≥ 0.700) of connectivity in PPI network were obtained (Figure 4). Download the tsv file in STRING. Then, the tsv file was used to analyzed PPI network by using Cytoscape v3.7.2.

### 3.4. Topological Analysis and Cluster Analysis

**Figure 5:**
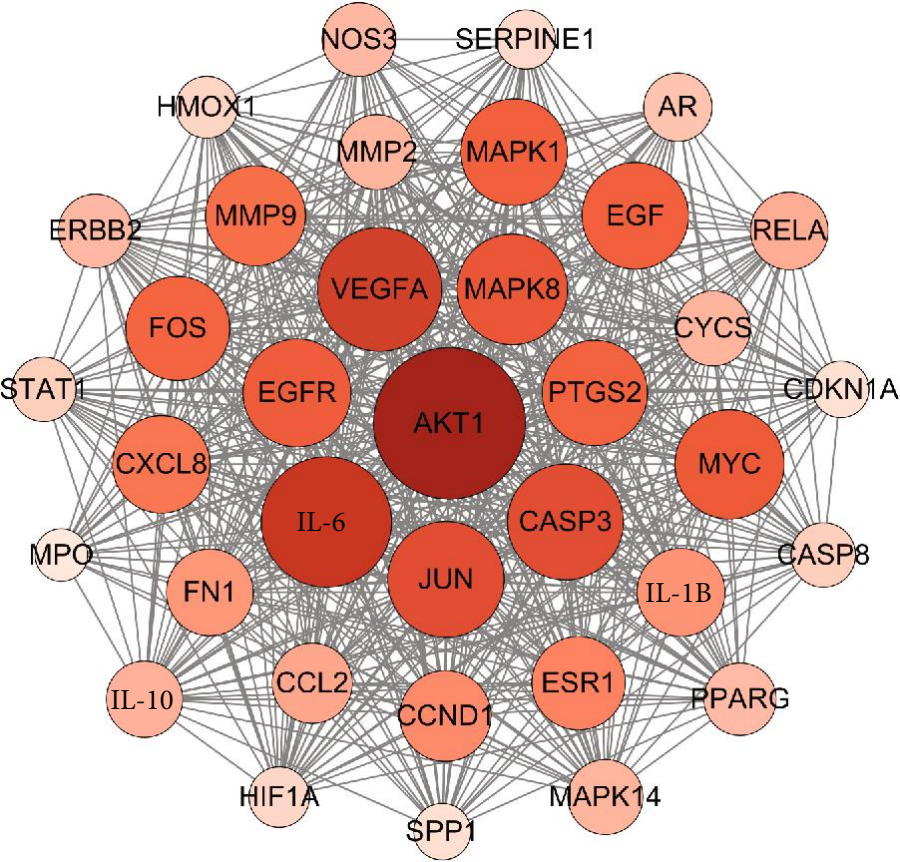
Key targets of HLJDD against T2DM.

The central properties of each node in PPI network were estimated by topological analysis. According to the network pharmacology method, based on criteria of DC ≥ 52, BC ≥ 71.46, CC ≥ 0.50, and EC ≥ 0.04, 39 key targets of HLJDD acting on T2DM were obtained (Table 3). In Figure 5, the larger of nodes was proportional to DC. Notably, AKT serine/threonine kinase 1 (AKT1, degree = 126) which played an essential role in the pathogenesis of T2DM was identified as the most important target in the PPI network. With regard to the target, some nodes (IL-6, VEGFA, MAPK8, JUN, CASP3, EGFR, PTGS2, and FOS) also have higher degrees, which were also recognized as important T2DM targets.

**Figure 6:**
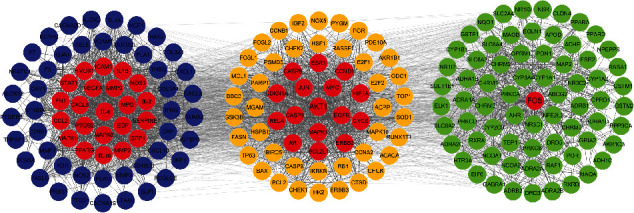
PPI network of target genes based in different cluster. Clusters 1, 2, and 3 are represented by dark blue, orange, and green, respectively. Red nodes represent the key targets. And the lines among nodes display the relationship between different target genes.

Community cluster (Cluster Marker tool) is a complex algorithm based on clustering objects with similar attributes. Target gene information in PPI network obtained from the STRING database was analyzed by Cluster Marker tool returned 3 central gene clusters. Clusters 1, 2, and 3 involved 70, 67, and 58 target proteins, respectively. In PPI network of target genes based on the results of cluster analysis and topology analysis, key targets are shown in red (Figure 6).

### 3.5. GO Functional Annotation Analysis

**Figure 7:**
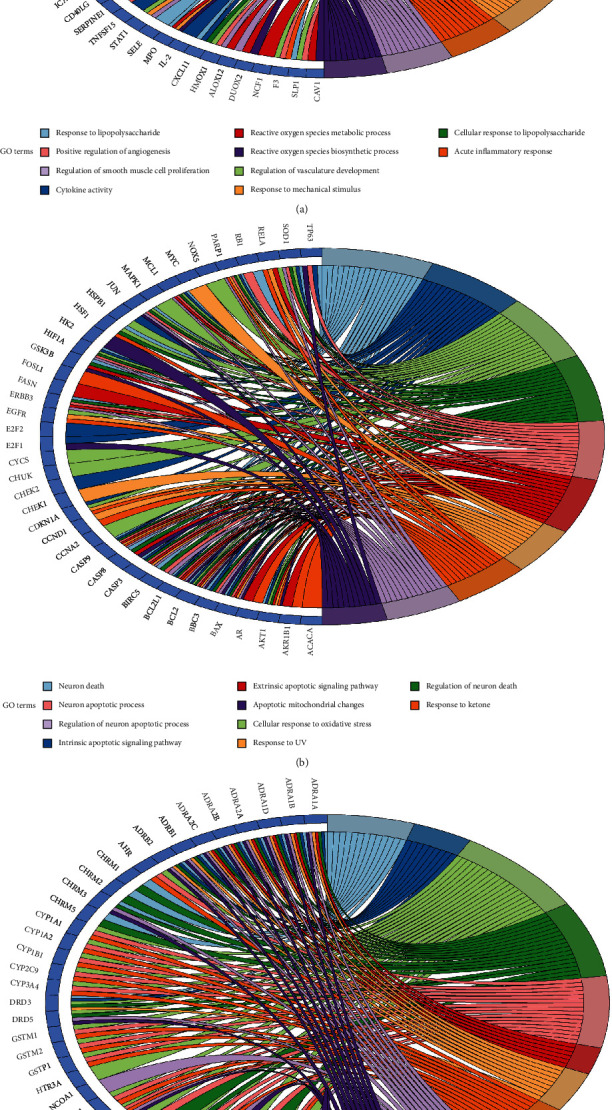
(a) GO biological process analysis of cluster 1. (b) GO biological process analysis of cluster 2. (c) GO biological process analysis of cluster 3.

ClueGO tool was used to perform GO and KEGG enrichment analyses on target genes in the above mentioned three modules. We studied the role of clusters 1, 2, and 3 in gene function and obtained 123, 137, and 119 GO entries, respectively, which were primarily involved in cell response to different stimuli, inflammatory response, regulation of apoptotic process, and regulation of apoptotic-related pathways, receptor activity, and vascular processes (such as angiogenesis, vasculature development, endothelial cell proliferation, nitric oxide biosynthesis, regulation of arterial blood pressure, vessel diameter, and vascular processes). The top 20 entries (*P* < 0.01) were selected based on *p* value and number of genes, displayed in Figure 7.

### 3.6. KEGG Pathway Analysis

**Figure 8:**
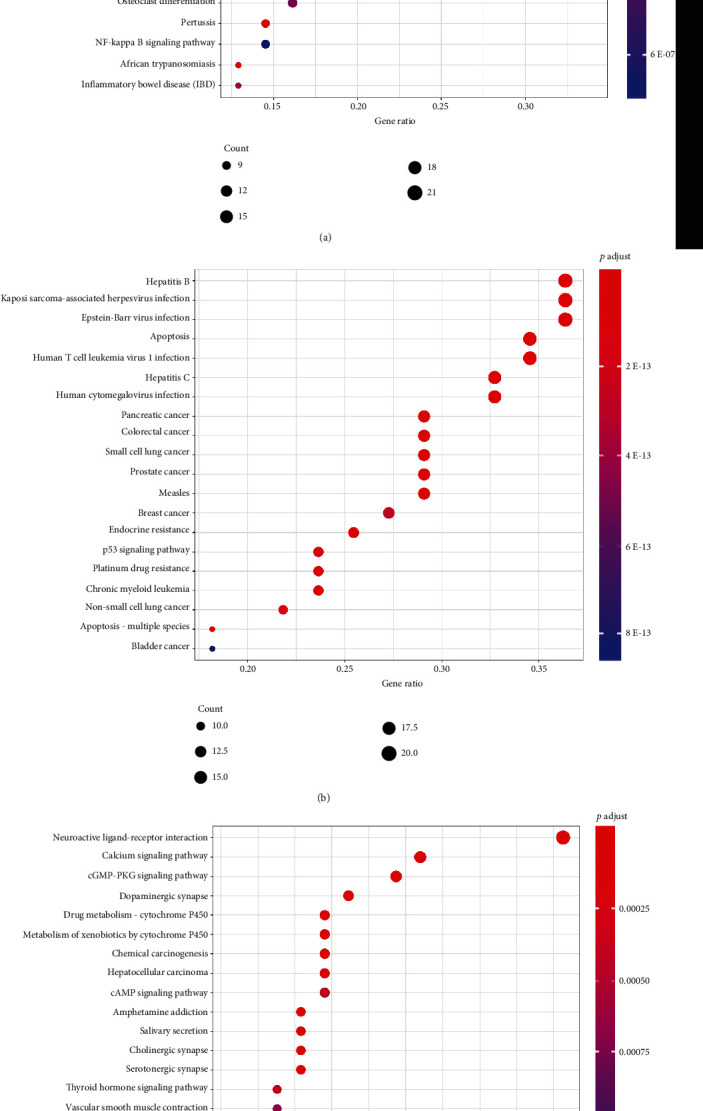
(a) KEGG pathway analysis of cluster 1. (b) KEGG pathway analysis of cluster 2. (c) KEGG pathway analysis of cluster 3.

KEGG pathway analysis shows that targets of different clusters were primarily distributed in pathways are related to diabetes and its complications. The representative top 20 pathways based on the number of enriched genes as well as *p* value are shown in Figure 8. Of these, cluster 1 mainly included AGE-RAGE signaling pathway in diabetic complications (*p* = 5.30E^−25^), fluid shear stress and atherosclerosis (*p* = 7.46E^−19^), TNF signaling pathway (*p* = 6.04E^−15^), and IL-17 signaling pathway (*p* = 1.30E^−14^). Module 2 focuses on apoptosis (*p* = 9.71E^−19^) and tumors pathways, including pancreatic cancer (*p* = 1.07E^−18^) and colorectal cancer (*p* = 6.55E^−18^). It is also involved in viral infections, such as hepatitis B (*p* = 9.71E^−19^), EB virus (*p* = 2.25E^−17^), and hepatitis C (*p* = 8.18E^−17^). In addition, endocrine resistance (*p* = 1.85E^−15^), insulin signaling pathway (*p* = 1.04E^−06^), insulin resistance (*p* = 1.91E^−04^), type II diabetes (*p* = 5.23E^−04^), and other signaling pathways also involved. Module 3 is related to neuroactive ligand-receptor interaction (*p* = 7.44E^−10^) and calcium signaling pathway (*p* = 1.63E^−07^), as well as the establishment of synapses, such as dopaminergic synapse (*p* = 1.22E^−06^), cholinergic synapse (*p* = 3.69E^−05^), and serotonergic synapse (*p* = 4.10E^−05^).

### 3.7. Differentially Expressed Analysis of AKT1, IL-6, and FOS in Human Type 2 Diabetic Visceral Adipose

**Figure 9:**
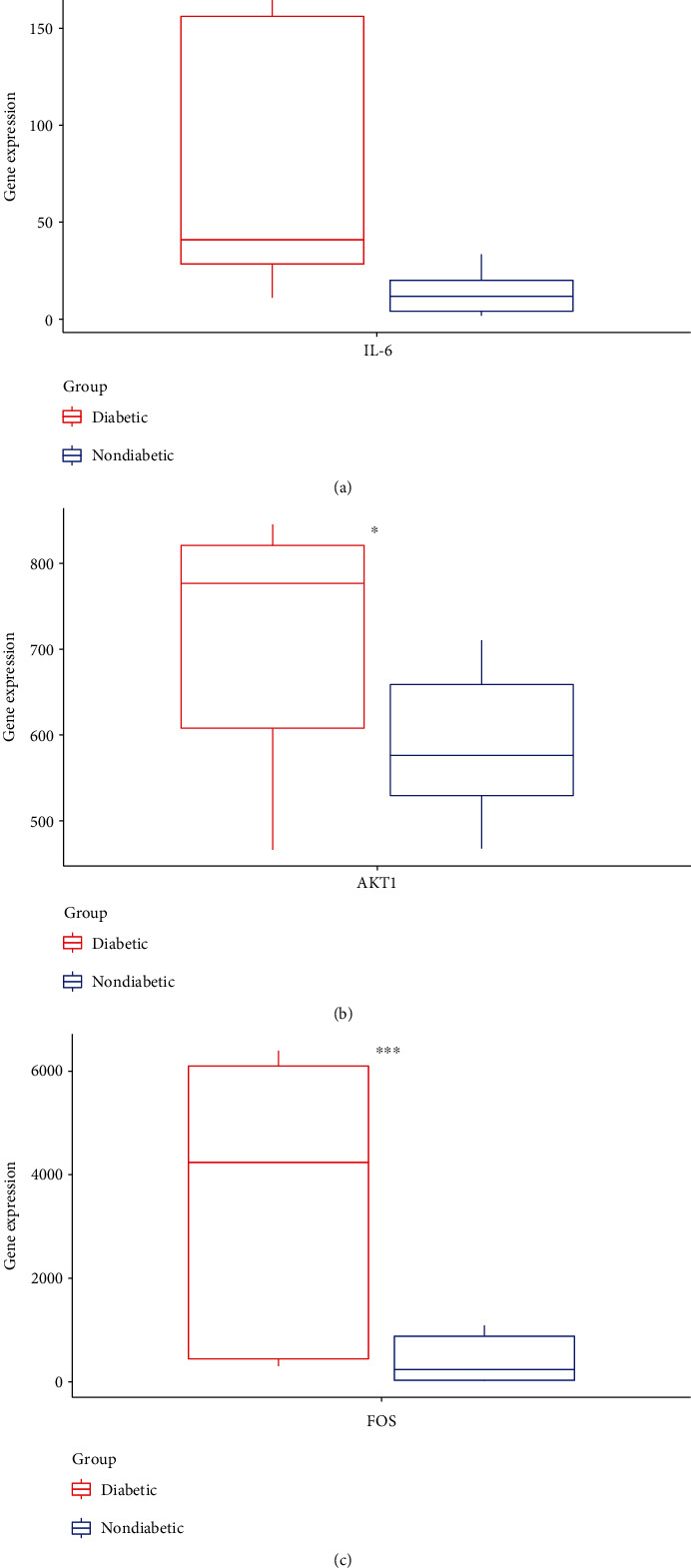
(a) IL-6. (b) AKT1. (c) FOS.

Gene expression data were downloaded from the GEO database, including 12 T2DM samples and 12 nondiabetic visceral adipose female controls from GSE29231. Differentially expressed analysis showed that AKT1, IL-6, and FOS were upregulated in T2DM samples, and a significant between sample differential expression with a *p* -value < 0.05. The results of gene expression in T2DM and nondiabetic samples are shown in Figure 9.

### 3.8. Validation of Compound-Target Interaction by Molecular Docking

To further validate the binding capacity between active compounds and key targets and improving the accuracy of target network, molecular docking through AutoDock was performed. Molecular docking analyzed three targets (AKT1, IL-6, and FOS) with highest DC in different clusters and 5 compounds (quercetin, wogonin, baicalein, kaempferol, oroxylin A) obtained from active components- targets network. The results are shown in Table 4. The binding energy values of most of them were smaller than -5 kJ∙mol^−1^, which showed that they possessed good binding activity.

### 3.9. Integrated Network Construction

**Figure 10:**
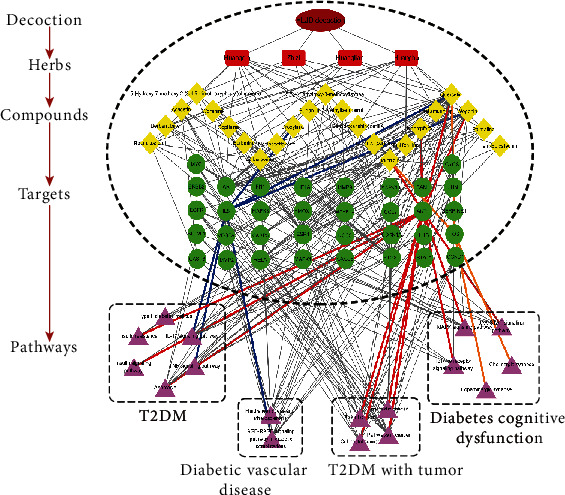
Decoction–compound–target–pathway network (Crimson indicates the HLJD decoction; red indicates the herbs; yellow indicates the compounds; green indicates the key targets; purple indicates the pathways).

To shed light on the potential mechanisms of HLJD decoction acting on T2DM, we constructed a decoction-compound-target-pathway network (Figure 10). The blue line is mainly connected to the diabetic vascular disease-related pathway—the pathway with the highest significance of *p* value and its key target-component complex with the highest binding energy values. The red line is connected to the T2DM, cancer, and diabetes cognitive dysfunction-related pathway—its key target-component complex with the highest binding energy values. The orange line is linked to the key target in the diabetes cognitive dysfunction-related pathway and the key target-component complex with the highest binding energy values. As observed in the results of network analysis, our research directly confirmed that multitarget of four herbs in HLJD decoction act synergistically in treating T2DM and its complications.

## 4. Discussion

Diabetes belongs to the category of “Xiao Ke” in TCM. It is roughly divided into three types, such as yin deficiency and dryness type, qi and yin deficiency type, and yin and yang deficiency type. Bitter-taste Chinese materia medica (CMM) is mainly used for the treatment of diabetes in yin deficiency and heat type [13]. In ancient times, there was a saying that “bitter can restrict sweetness,” and there are records in ancient books of using CMM to treat diabetes, because “strengthening Yin with bitter-flavor herbs,” which means that some CMMs can protect the yin by reducing heat [14]. HL, HQ, HB, and ZZ in HLJDD formula are all CMM and have the effect of clearing away heat. According to the published literature, HLJDD has exact prevention and treatment effect on diabetes and its complications [15–18].

In the present study, we applied the systematic pharmacological method to predict and elucidate the potential molecular mechanisms of action of the HLJDD on T2DM. In the HLJDD's active components-targets network, a total of 203 targets affected by 66 bioactive compounds in the HLJDD were obtained. There were 4 compounds including quercetin, wogonin, kaempferol, and baicalein identified as the potential active ingredients of HLJDD, of which the biological activities against T2DM were reported previously. For example, quercetin and kaempferol, wogonin, and baicalein, that is, are flavonoid compounds with a similar mechanism of action as metformin, which could reduce glucose and improve glucose Glut4 and AMPK expression in skeletal muscle and adipose tissue, thereby increasing insulin sensitivity and improvement of islet *β* cell quality [19–21]. Alkhalidy illustrated that kaempferol can increase AKT and hexokinase activity, reduce the activity of pyruvate carboxylase and glucose-6 phosphatase in the liver, and exert antidiabetic effects by inhibiting gluconeogenesis in the liver [22]. In addition, kaempferol also can reduce myocardial ischemia-reperfusion injury/MAPK-induced oxidative stress and inflammation by reducing AGE-RAGE, thereby improving myocardial injury in diabetic rats [23]. Yang discovered that baicalein (10^−6^ and 10^−5^ mol/L) may promote glucose uptake and glycolysis through the InsR/IRS-1/PI3K/AKT pathway and inhibit gluconeogenesis in liver cells, thus have strong anti-IR hepatocyte activity [24]. Therefore, the above active components indicate the effectiveness and diversity of chemical ingredients in HLJDD for treating T2DM. In addition, oroxylin A also plays an important role in the network, oroxylin A possessing a broad spectrum of pharmacological effects, especially anticancer, anti-inflammatory, and neuroprotective activity [25], but there is relatively little research on its pharmacological treatment of T2DM, which deserves further discussion.

The results of PPI network analysis acquired 197 targets of HLJDD acting on T2DM. The GO enrichment analysis of 197 targets indicates that the therapeutic effect of the HLJDD in T2DM mainly involved in biological processes such as cell response to different stimuli, regulation of transcription, inflammatory response, regulation of apoptotic process and regulation of apoptotic-related pathways, receptor activity, vascular processes, and regulation of gene expression, via molecular function of protein binding. Apoptosis plays important roles in the pathophysiology of T2DM [26]. Rehab observed that the STZ-induced diabetes significantly increased the expression of the apoptosis biomarker C-fos gene [27]. Experimental studies have also confirmed that MYC, CASP3, JUN, and EGFR are related to islet *β*-cell apoptosis [28], while AKT1 acts as an antiapoptotic signaling kinase in a variety of cells. Zhang Rui found that increasing the expression of Akt in islet cells can reduce islet cell apoptosis and increase secretory function [29]. In addition, Xu have found that AKT1 gene was activated CpG island demethylation in the promoter of the AKT1 gene is involved in the occurrence of cardiovascular and cerebrovascular complications in T2DM [30]. IL-6 has been identified as a key mediator of inflammation, immune response, and glucose metabolism; it is the basis of insulin resistance in patients with T2DM [31]. Wu et al. discovered that tocilizumab (an IL-6 receptor antibody) inhibits the activation of NLRP3 inflammasome by inhibiting IL-17A, improves insulin resistance, and has protective effects on diabetic kidney injury [32]. In addition to being directly related to T2DM, the polymorphisms of VEGFA, EGFR, PTGS2, and AKT1 genes are mainly related to diabetic vascular dysfunction and play an important role in diabetic vascular disease and diabetic nephropathy [33–36].

As observed in the results of KEGG enrichment analysis, some signaling pathways (such as AGE-RAGE signaling pathway in diabetic, IL-17signaling pathway, TNF signaling pathway, Toll-like receptor signaling pathway, type II diabetes, endocrine resistance, and insulin resistance signaling pathways) are closely related to the development and metastasis of T2DM. Increasing evidence indicates that chronic hyperglycemia leads to the formation of AGEs, and AGE-RAGE activation leads to oxidative stress and inflammation associated with cardiovascular complications of diabetes [23, 37]. There is also accumulation of evidence that AGE/RAGE interacts with TNF signaling pathway, perhaps even through mutual amplification to induce the production of superoxide in T2DM [38]. Fluid shear stress and flow pattern also play an important role in atherosclerosis [39]. Deng et al. found that HLJDD can significantly reduce blood glucose and glycosylated hemoglobin (GH) in diabetic rats and inhibit the generation of AGEs in plasma and kidney tissues (*p* < 0.01) [40].

Some experts had put forward some hypothesis that insulin resistance, inflammation, accumulation of advanced glycation end products (AGEs), and oxidative stress may be associated with an increased risk of dementia in the T2DM population [41, 42]. It is reported that HLJDD plays an important role in the mechanism of neuronal injury through neuroinflammation-related signaling pathways, such as MAPK, Toll-like receptors, and JNK/SAPK9 pathways, what is more, it can also act on the insulin signaling pathway to clear/reduce A*β* in the brain and inhibit Tau protein hyperphosphorylation [43]. HLJDD can improve diabetes cognitive dysfunction by regulating neuroinflammation-related signaling pathways in cluster 2. Acetylcholine was significantly reduced in diabetic patients [44]. It is found that HLJDD has effects on neurotransmitters and regulatory pathways such as cholinergic, serotonergic, and dopaminergic synapses in cluster 3.

In cardiomyocytes, Ca^2+^ is the core of excitation-contraction coupling and affects various signal cascades [45]. In the pathogenesis of diabetic cardiomyopathy, AGEs stimulated the increase of Ca^2+^ and reduces MMP, which eventually damages myocardial contractile and diastolic functions, and induces apoptosis [46]. HLJDD prevents and treats diabetic cardiomyopathy via calcium signaling pathway in cardiomyocytes in cluster 3.

Many studies supporting that hyperglycemia, insulin resistance, hyperinsulinemia, IGF-1 levels, dyslipidemia, inflammatory, and cytokines affect tumor cell microenvironment and intracellular signal transduction and help tumor growth and progression [47]. The overall assessment of many large-scale epidemiological studies and meta-analyses has shown that the incidence of cancer at specific sites in T2DM patients continues to increase, including pancreatic cancer, hepatobiliary cancer, breast cancer, and colorectal cancer [47]. HLJDD exhibits antitumor effect involved in regulating multiple tumor-related pathways in cluster 2.

The top degrees of the potential target genes in the three clusters for the HLJDD in the treatment of T2DM by cluster and topological analysis include AKT1, IL-6, and FOS. We compared the gene expression levels of AKT1, IL-6, and FOS between diabetic patients and nondiabetic and the statistical relationship. The analysis results of differential expression verified that AKT1, IL-6, and FOS were upregulated in T2DM samples, and the difference between the samples was significant (*p* < 0.05). Further molecular docking assay in this study showed that quercetin was verified strong binding activity with AKT1, IL-6, and FOS, while baicalein had strong combination with AKT1 and FOS, wogonin had strong combination with AKT1 and IL-6, kaempferol had strong combination with AKT1, and oroxylin A had strong combination with IL-6.

## 5. Conclusion

In conclusion, this comprehensive bioinformatic analysis provides numerous testable hypotheses about the molecular mechanisms of HLJDD formula for the treatment of T2DM as well as diabetic complications (cognitive dysfunction, vascular disease, and cardiomyopathy) and predicted that quercetin, baicalein, wogonin, kaempferol, and oroxylin A were potential active ingredients of HLJDD, which acts on key genes such as AKT1, IL-6, and FOS to regulate many biological processes (signaling transduction, inflammatory response, apoptotic process, vascular processes, etc.) and pathways (AGE-RAGE signaling pathway in diabetic, IL-17signaling pathway, TNF signaling pathway, Toll-like receptors signaling pathway, type II diabetes, endocrine resistance, insulin resistance signaling pathways, etc.). It also should be noted that HLJDD formula possible exhibits antitumor effect by regulating multiple tumor-related pathways. The prediction results provided the theoretic elucidation of the ameliorative effect of HLJDD against T2DM and might facilitate the development of HLJDD or its active compounds as alternative therapy for T2DM.

## Figures and Tables

**Table 1:** Database and website.

Database	Website
TCMSP	http://lsp.nwu.edu.cn/tcmsp.php
Uniprot	http://www.uniprot.org
GeneCards	https://www.genecards.org/
OMIM	https://www.omim.org
Venny	http://bioinfogp.cnb.csic.es/tools/venny/index.html
STRING	https://string-db.org/
Omishare	http://www.omicshare.com/tools/index.php/
RCSB PDB	https://www.rcsb.org
PubChem Compound	https://pubchem.ncbi.nlm.nih.gov
GEO	https://www.ncbi.nlm.nih.gov/geo/

**Table 2:** Active components network information.

Mol ID	Compound	OB (%)	DL	Herb
MOL002915	Salvigenin	49.07	0.33	HQ
MOL002934	NEOBAICALEIN	104.34	0.44	HQ
MOL000173	Wogonin	30.68	0.23	HQ
MOL002937	DIHYDROOROXYLIN	66.06	0.23	HQ
MOL002927	Skullcapflavone II	69.51	0.44	HQ
MOL002897	Epiberberine	43.09	0.78	HQ, HL
MOL002925	5,7,2′,6′-Tetrahydroxyflavone	37.01	0.18	HQ
MOL002932	Panicolin	76.26	0.29	HQ
MOL000228	(2R)-7-Hydroxy-5-methoxy-2-phenylchroman-4-one	55.23	0.20	HQ
MOL002910	Carthamidin	41.15	0.24	HQ
MOL002714	Baicalein	33.52	0.21	HQ
MOL002913	Dihydrobaicalin_qt	40.04	0.21	HQ
MOL002914	Eriodyctiol (flavanone)	41.35	0.24	HQ
MOL002928	Oroxylin a	41.37	0.23	HQ
MOL002933	5,7,4′-Trihydroxy-8-methoxyflavone	36.56	0.27	HQ
MOL000449	Stigmasterol	43.83	0.76	HQ, ZZ, HB
MOL002909	5,7,2,5-Tetrahydroxy-8,6-dimethoxyflavone	33.82	0.45	HQ
MOL001689	Acacetin	34.97	0.24	HQ
MOL001458	Coptisine	30.67	0.86	HQ, HL, HB
MOL002917	5,2′,6′-Trihydroxy-7,8-dimethoxyflavone	45.05	0.33	HQ
MOL000552	5,2′-Dihydroxy-6,7,8-trimethoxyflavone	31.71	0.35	HQ
MOL000073	ent-Epicatechin	48.96	0.24	HQ
MOL000359	Sitosterol	36.91	0.75	HQ
MOL000358	Beta-sitosterol	36.91	0.75	HQ, ZZ, HB
MOL012245	5,7,4′-Trihydroxy-6-methoxyflavanone	36.63	0.27	HQ
MOL010415	11,13-Eicosadienoic acid, methyl ester	39.28	0.23	HQ
MOL008206	Moslosooflavone	44.09	0.25	HQ
MOL002879	Diop	43.59	0.39	HQ
MOL012266	Rivularin	37.94	0.37	HQ
MOL000525	Norwogonin	39.40	0.21	HQ
MOL001490	bis[(2S)-2-Ethylhexyl]	43.59	0.35	HQ
MOL002933	5,7,4′-Trihydroxy-8-methoxyflavanone	36.56	0.27	HQ
MOL002670	Cavidine	35.64	0.81	HB
MOL002666	Chelerythrine	34.18	0.78	HB
MOL002663	Skimmianin	40.14	0.20	HB
MOL002668	Worenine	45.83	0.87	HB, HL
MOL002662	Rutaecarpine	40.30	0.60	HB
MOL005438	Campesterol	37.58	0.71	HB
MOL000622	Magnograndiolide	63.71	0.19	HB, HL
MOL001771	Poriferast-5-en-3beta-ol	36.91	0.75	HB
MOL002644	Phellopterin	40.19	0.28	HB
MOL002643	Delta 7-stigmastenol	37.42	0.75	HB
MOL000790	Isocorypalmine	35.77	0.59	HB
MOL000787	Fumarine	59.26	0.83	HB
MOL001131	Phellavin_qt	35.86	0.44	HB
MOL006422	Thalifendin	44.41	0.73	HB
MOL001131	Phellamurin_qt	56.60	0.39	HB
MOL001455	(S)-Canadine	53.83	0.77	HB
MOL000785	Palmatine	64.60	0.65	HB, HL
MOL002651	Dehydrotanshinone II A	43.76	0.40	HB
MOL003095	5-Hydroxy-7-methoxy-2-(3,4,5-trimethoxyphenyl) chromone	51.96	0.41	ZZ
MOL001494	Mandenol	42.00	0.19	ZZ
MOL007245	3-Methylkempferol	60.16	0.26	ZZ
MOL001942	Isoimperatorin	45.46	0.23	ZZ
MOL002883	Ethyl oleate (NF)	32.40	0.19	ZZ
MOL004561	Sudan III	84.07	0.59	ZZ
MOL001406	Crocetin	35.30	0.26	ZZ
MOL000422	Kaempferol	41.88	0.24	ZZ
MOL001941	Ammidin	34.55	0.22	ZZ
MOL002907	Corchoroside A_qt	104.95	0.78	HL
MOL002904	Berlambine	36.68	0.82	HL
MOL002894	Berberrubine	35.74	0.73	HL, HB
MOL000098	Quercetin	46.43	0.28	HL, ZZ, HB
MOL001454	Berberine	36.86	0.78	HL, HB
MOL002903	(R)-Canadine	55.37	0.77	HL

**Table 3:** The 39 key targets of HLJDD in treating T2DM.

Target symbol	Target name	DC	BC	CC	EC
PTGS2	Prostaglandin G/H synthase 2	91	1047	0.640	0.149
MPO	Myeloperoxidase	91	203	0.553	0.091
CCL2	C-C motif chemokine 2	92	261	0.595	0.130
EGF	Proepidermal growth factor	92	1100	0.640	0.147
NOS3	Nitric oxide synthase, endothelial	91	261	0.593	0.106
IL-10	Interleukin-10	91	261	0.582	0.130
SPP1	Osteopontin	91	261	0.561	0.104
EGFR	Epidermal growth factor receptor	93	958	0.642	0.145
CCND1	G1/S-specific cyclin-D1	92	401	0.610	0.135
CDKN1A	Cyclin-dependent kinase inhibitor 1	91	261	0.556	0.135
JUN	Transcription factor AP-1	99	1005	0.655	0.158
MAPK14	Mitogen-activated protein kinase 14	91	261	0.584	0.135
HIF1A	Hypoxia-inducible factor 1-alpha	91	261	0.562	0.135
MMP2	72 kDa type IV collagenase	91	261	0.586	0.135
CASP3	Caspase-3	99	1335	0.658	0.156
HMOX1	Heme oxygenase 1	91	261	0.567	0.135
PPARG	Peroxisome proliferator activated receptor gamma	91	261	0.586	0.135
IL-2	Interleukin-2	91	261	0.562	0.135
FOS	Proto-oncogene c-Fos	91	1828	0.640	0.137
SERPINE1	Plasminogen activator inhibitor 1	91	261	0.566	0.135
MAPK8	Mitogen-activated protein kinase 8	95	788	0.649	0.154
FN1	Fibronectin	92	473	0.604	0.135
CASP8	Acacetin	91	261	0.564	0.135
CYCS	Cytochrome c	91	261	0.584	0.135
ERBB2	Receptor tyrosine-protein kinase erbB-2	91	261	0.590	0.135
BCL2L1	Bcl-2-like protein 1	91	261	0.569	0.135
AR	Androgen receptor	91	261	0.584	0.135
MYC	Myc proto-oncogene protein	94	724	0.642	0.149
MMP9	Matrix metalloproteinase-9	91	1088	0.626	0.147
MAPK1	Mitogen-activated protein kinase 1	92	988	0.638	0.150
IL-1B	Interleukin-1 beta	92	397	0.610	0.135
CXCL8	Interleukin-8	91	674	0.622	0.142
ICAM1	Intercellular adhesion molecule 1	91	261	0.579	0.135
VEGFA	Vascular endothelial growth factor A	105	1104	0.664	0.164
AKT1	RAC-alpha serine/threonine-protein kinase	126	3090	0.729	0.174
RELA	Transcription factor p65	92	261	0.588	0.135
IL-6	Interleukin-6	110	1654	0.683	0.163
ESR1	Estrogen receptor	91	741	0.616	0.131
STAT1	Signal transducer and activator of transcription 1-alpha/beta	91	261	0.567	0.135

**Table 4:** Docking score of active compounds with key targets of HLJD decoction.

Protein	PDB ID	Compounds	Molecular formula	Structure	3D molecular docking diagrams	H-bond	Binding energy /(kJ∙Mol^−1^)
IL-6	6AE3	Oroxylin A	C_16_H_12_O_5_	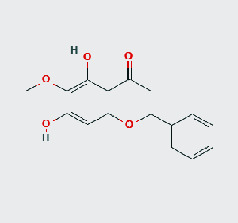	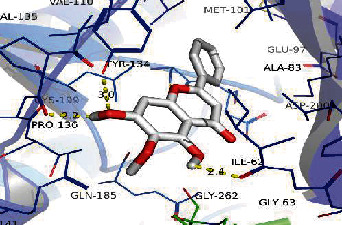	PRO136 TYR134 ILE62	-35.53
		Quercetin	C_15_H_10_O_7_	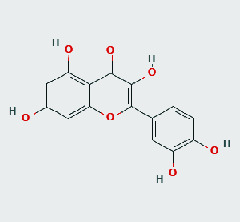	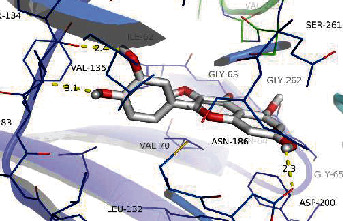	VAL135 ASP200	-34.28
		Wogonin	C_16_H_12_O_5_	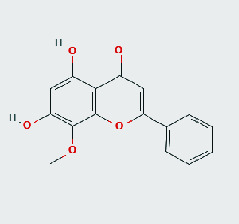	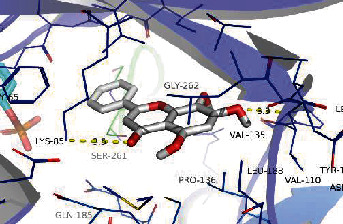	LYS85 VAL135	-34.69

AKT1	6HHI	Baicalein	C_15_H_10_O_5_	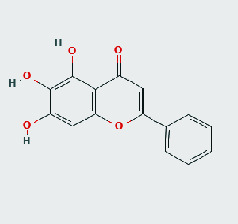	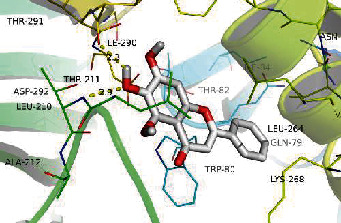	THR211 ILE290	-38.87
		Kaempferol	C_15_H_10_O_6_	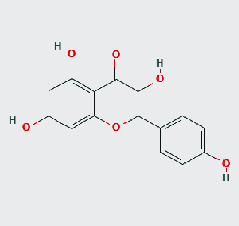	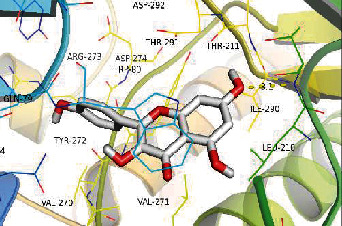	THR211	-36.78
		Quercetin	C_15_H_10_O_7_	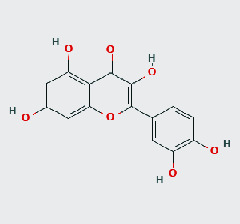	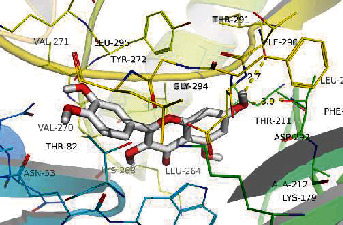	THR211 ILE290	-37.62
		Wogonin	C_16_H_12_O_5_	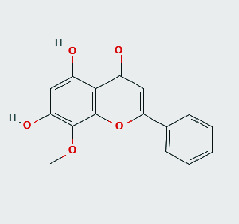	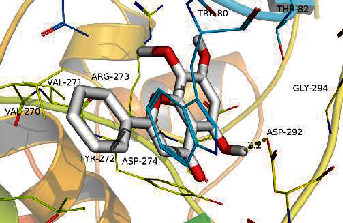	ASP292	-37.20

FOS	4 L13	Baicalein	C_15_H_10_O_5_	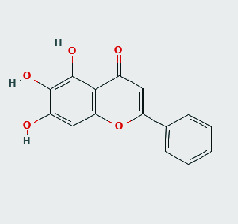	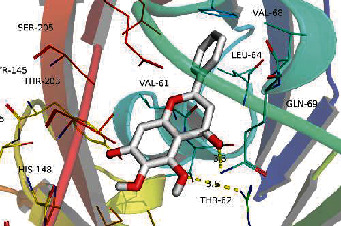	GLN69 ARG96	-25.50
		Quercetin	C_15_H_10_O_7_	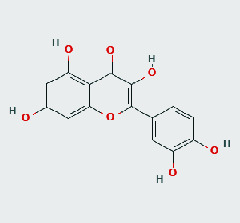	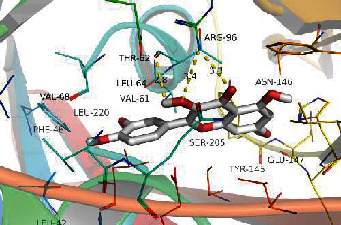	ARG96 THR62	-19.26

## Data Availability

The data used to support the findings of this study are available from the corresponding author upon request.
